# Posttraumatic stress disorder, depression, and non-fatal intentional self-harm in Massachusetts Veterans

**DOI:** 10.1186/s40621-014-0020-5

**Published:** 2014-08-27

**Authors:** Jaimie L Gradus, Sarah Leatherman, Sanjay Raju, Ryan Ferguson, Matthew Miller

**Affiliations:** 1 National Center for PTSD, VA Boston Healthcare System, Boston, USA; 2 Boston University, Boston, USA; 3 Massachusetts Veterans Epidemiology Research and Information Center, VA Boston Healthcare System, Boston, USA; 4 Department of Health Policy and Management, Harvard School of Public Health, Boston, USA

**Keywords:** Stress disorder, Post-traumatic, Deliberate self-harm, Veterans

## Abstract

**Background:**

The literature on the association between Posttraumatic Stress Disorder (PTSD) and fatal and non-fatal intentional self-harm (ISH) among Veterans who receive care within the Veterans Health Administration (VHA) is limited in scope and contradictory. The current study examines the association between PTSD and non-fatal ISH in a gender-stratified sample of patients who received care at a Massachusetts VHA treatment facility between 2000 and 2008.

**Methods:**

VHA electronic medical record data were obtained for patients who received a PTSD diagnosis at a Massachusetts treatment facility (n = 16,004) and a gender/age matched comparison group (n = 52,502). Rate ratios for the association between PTSD and non-fatal ISH were computed adjusting for marital status, depression, alcohol or drug abuse or dependence, anxiety disorder diagnoses and prior ISH and clustering by hospital using Poisson regression. The interaction between PTSD and depression diagnoses in predicting non-fatal ISH was assessed as the departure from additive effects by calculating the interaction contrast (IC) while adjusting for identified confounders.

**Results:**

Over the eight year study period 146 (0.91%) of those with PTSD experienced non-fatal ISH, while 71 (0.14%) of those without PTSD experienced non-fatal ISH. Strong adjusted associations between PTSD and non-fatal ISH were found for both male (RR = 3.3, 95% CI = 2.3, 4.6) and female (RR = 16, 95% CI = 4.7, 55) VHA patients. Evidence of an interaction between PTSD and depression diagnoses in predicting non-fatal ISH was found as a departure from additive effects for both sexes, but this association was more marked among women than among men.

**Conclusions:**

Our results indicate that non-fatal ISH among women may be more strongly related to PTSD than prior work focusing on suicide has suggested and highlight the importance of gender-stratified examinations of these associations. Further, our results suggest that suicide prevention approaches in the VHA should integrate treatment for PTSD and depression.

Posttraumatic Stress Disorder (PTSD) is a well-established risk factor for suicidal ideation, non-fatal and fatal self-harm in the general population (Gradus et al. [2010]; Sareen et al. [2005]; Davidson et al. [1991]; Wilcox et al. [2009]; Sareen et al. [2007]) and among Veterans in general (Bullman and Kang [1994]; Kramer et al. [1994]; Boscarino [2006]; Panagioti et al. [2009]; Pompili et al. [2013]; Gradus et al. [2013]; Jakupcak et al. [2009]; Pietrzak et al. [2010]; Lemaire and Graham [2010]). In contrast, the literature on the association between PTSD and both fatal and non-fatal intentional self-harm (ISH) among Veterans who receive care within the Veterans Health Administration (VHA) is contradictory and limited in scope (Ilgen et al. [2010]; Zivin et al. [2007]; Brenner et al. [2011]; Drescher et al. [2003]).

The only study of which we are aware to examine PTSD as a predictor of non-fatal ISH among VHA patients found that patients with PTSD had nearly three times the odds of ISH compared with patients without PTSD, adjusting for traumatic brain injury only (Brenner et al. [2011]). Given the paucity of literature on the association between PTSD and non-fatal self-harm specifically among VHA patients, the literature on PTSD and death from suicide in this population may provide additional context for future research on non-fatal self-harm. One small study found that male VHA patients in residential treatment for PTSD were four times as likely to die by suicide as were age and sex-matched members of the general population (Drescher et al. [2003]). The most recent and largest study of VHA patients, and the only study to stratify by sex, found that among VHA patients diagnosed with PTSD in 1999, males were 1.8 times as likely and females were 3.5 times as likely to die by suicide in the seven years that followed than were patients without a PTSD diagnosis, while adjusting for age but not other potential confounders such as psychiatric comorbidity (Ilgen et al. [2010]). In contrast to these findings, Zivin et al. ([2007]) found that a PTSD diagnosis was protective against suicide among VHA patients with depression (rate ratio = 0.78; 95% confidence interval = (0.70, 0.87)) (Zivin et al. [2007]). This work also contradicts studies of the general population have found that PTSD increases risk for suicide attempts among adults with depression, (Stevens et al. [2013]) and that comorbid PTSD and depression are associated with a greater risk of suicide attempts (compared to having just one of these diagnoses) in clinical samples (Oquendo et al. [2003]; Oquendo et al. [2005]).

The current study is the first of which we are aware to assess separately for male and female VHA patients 1) the association between PTSD and non-fatal ISH in a large, longitudinal sample while adjusting for several potential confounders, and 2) the interaction between PTSD and depression in predicting non-fatal ISH.

## Methods

### Study population

The base population consists of patients who received care at a Massachusetts VHA treatment facility between fiscal years 2000 and 2008. Patients were considered to be a VHA health care user if they had at least one primary care and one other health care visit in the twelve months prior their PTSD diagnosis or index date for comparison group members (comparison group members were chosen as a match for a PTSD participant if they received care at a Massachusetts VA in the same year that a PTSD participant was diagnosed. The index date for the comparison group member is the date of their matched cases PTSD diagnosis). The twelve month time frame also permitted equal ascertainment time of baseline characteristics for those with PTSD and the comparison group.

The records of patients who received a PTSD diagnosis (ICD-9-CM code 309.81) at a Massachusetts VHA treatment facility between fiscal years 2000 and 2008 were obtained from the electronic medical record (n = 16,004). A comparison cohort of veterans who received care at a Massachusetts VHA treatment facility, but who never received a PTSD diagnosis, were matched to PTSD patients, with a ratio of up to 5 to 1, on five-year age categories and gender (n = 52,502). Data were obtained for the following variables during the study period: depression diagnoses (ICD-9-CM codes: 296.2-296.3), substance use disorders (ICD-9-CM codes: 303.xx-305.9), anxiety disorders (ICD-9-CM codes: 300, 300.01-300.02), and ISH resulting in an inpatient hospitalization (ICD-9-CM codes: E950-E959).

### Statistical analyses

Descriptive analyses were conducted to characterize Veterans with and without a PTSD diagnosis with regard to demographic variables, depression diagnoses, substance use disorders, anxiety disorders, and prior ISH.

A Veteran’s person-time was measured from date of PTSD diagnosis (or the index date for comparison group members) to the first non-fatal ISH episode resulting in hospitalization, death, or end of the last fiscal year in which a patient fulfilled the study definition of a VA health care user, whichever came first. Unadjusted rate ratios and corresponding confidence intervals were calculated to compare the rate of non-fatal ISH among veterans with and without PTSD. Rate ratios were then computed adjusting for marital status, depression, alcohol or drug abuse or dependence, anxiety disorder diagnoses, and prior ISH and clustering by hospital (with each of the 25 facilities in MA represented by a numerical code) using Poisson regression. Confounders selected for adjustment in the regression analyses were based on the literature, and restricted to the time period before PTSD diagnosis (or index date for the comparison group). (Kessler et al. [1995]; Debell et al. in press). The interaction between PTSD and depression diagnoses (occurring at any point during the study period) in predicting non-fatal ISH was assessed as the departure from additive effects by calculating the interaction contrast (IC) while adjusting for identified confounders (Greenland et al. [2008]). All analyses were conducted using SAS v.9.2 (SAS. 9.2 [2009]). This study was approved by the Institutional Review Board at VA Boston Healthcare System.

## Results

The majority of participants were male and Caucasian (Table 1). Baseline psychiatric diagnoses (e.g., depression, alcohol and drug abuse/dependence, and anxiety disorder) and prior ISH were more common among Veterans who had been diagnosed with PTSD than among those who had not received a PTSD diagnosis. Non-fatal ISH events during the study period were also more frequent among people with PTSD (n = 146; 0.91%) than people without PTSD (n = 71; 0.14%). Further, among those with PTSD 25 (2.6%) women and 121 (0.80%) men had a non-fatal ISH event, while among those without PTSD 4 (0.09%) women and 67 (0.14%) men had a non-fatal ISH event. The average length follow up for those with PTSD was 4.1 years, while the average length of follow-up for those without PTSD was 3.6 years.

**Table 1:** **Demographics and comorbidities among Massachusetts VHA patients with PTSD and comparison group, 2000-2008**

	PTSD		Comparison group	
	(N = 16,004)		(N = 52,502)	
	Males	Females	Males	Females
	(N = 15,056)	(N = 948)	(N = 48,042)	(N = 4,460)
*Demographics*				
Race, n (%)				
White	12,872 (88.0%)	755 (83.0%)	36,599 (85.2%)	2,297 (68.3%)
Black	1,314 (9.0%)	126 (13.9%)	2,958 (6.9%)	306 (9.1%)
Asian	46 (0.3%)	6 (0.7%)	149 (0.4%)	42 (1.3%)
American Indian	30 (0.21%)	3 (0.3%)	106 (0.3%)	12 (0.4%)
Unknown	368 (2.5%)	20 (2.2%)	3,168 (7.4%)	705 (21.0%)
Marital status				
Married	6,800 (46.9%)	211 (23.3%)	22,267 (47.1%)	923 (20.8%)
Divorced	3,532 (24.4%)	286 (31.5%)	9,910 (21.0%)	593 (13.4%)
Never/Single	3,110 (21.4%)	333 (36.7%)	10,092 (21.3%)	796 (18.0%)
Widowed	659 (4.5%)	47 (5.2%)	3,068 (6.5%)	156 (3.5%)
Single	247 (1.7%)	14 (1.5%)	265 (0.6%)	18 (0.4%)
Unknown	157 (1.1%)	16 (1.8%)	1,708 (3.6%)	1,948 (43.9%)
Age, Mean (SD, range)	56.4 (13.8, 19.8 – 96.7)	44.3 (12.2, 19.4 – 85.5)	59.4 (15.1, 17.3 – 97.4)	44.8 (12.1, 16.7 – 89.6)
*Comorbidities*				
Depression diagnosis, n (%)	1,704 (11.3%)	175 (18.5%)	1,135 (2.4%)	117 (2.6%)
Alcohol abuse/dependence, n (%)	2,381 (15.8%)	97 (10.2%)	2,604 (5.4%)	49 (1.1%)
Drug abuse/dependence, n (%)	2,729 (18.1%)	153 (16.1%)	4,350 (9.1%)	162 (3.6%)
Anxiety disorders, n (%)	1,949 (13.0%)	132 (13.9%)	1,660 (3.5%)	124 (2.8%)
Prior suicide attempt, n (%)	44 (0.3%)	5 (0.5%)	8 (0.02%)	3 (0.07%)

Note. PTSD = posttraumatic stress disorder.

Among male Veterans the unadjusted association between PTSD and non-fatal ISH was 5.0 (95% confidence interval (CI): 3.7, 6.8). After adjusting for baseline marital status, depression, alcohol and drug abuse/dependence, anxiety disorder diagnoses and prior ISH this association was reduced to 3.2 (95% CI: 2.3, 4.5). For female Veterans, the association between PTSD and non-fatal ISH was 24 (95% CI: 8.2, 68). This association decreased to 16 (95% CI: 4.8, 56) after adjustment for baseline marital status, depression, alcohol and drugabuse/dependence, anxiety disorder diagnoses and prior ISH^a^.

Gender-stratified adjusted interaction contrasts for the association between PTSD and depression occurring any time during the study period in predicting non-fatal ISH (Table 2) reveal that among male VHA patients, there were 11.6 extra non-fatal ISH events among those with PTSD and depression per 100,000 person-years (py) that could not be explained by the individual effects of PTSD (82.6/100,000 py), depression (142.3/100,000 py) or background causes (i.e., all of the other causes of ISH that are neither PTSD nor depression) of non-fatal ISH (24.6/100,000 py). Women diagnosed with both PTSD and depression at any point during the study period experienced 747.7 additional cases of non-fatal ISH per 100,000 person-years than what would be expect based on the number of non-fatal ISH events among women with PTSD only (137.4/100,000 py), depression only (80.5/100,000 py) or the background causes of non-fatal ISH (25.0/100,000 py)^b^.

**Table 2:** **Adjusted incident rates and interaction contrast for intentional self-harm per 100,000 person years by PTSD and depression diagnoses in massachusetts VHA patients, 2000-2008**

Men		
	PTSD +	PTSD -
Depression +	211.9	142.3
Depression -	82.6	24.6
	aIC = 11.6	
Women		
	PTSD +	PTSD -
Depression +	940.6	80.5
Depression -	137.4	25.0
	aIC = 747.7	

Note: aIC = adjusted interaction contrast; PTSD = posttraumatic stress disorder.

Adjusted for: marital status, alcohol abuse or dependence, drug abuse or dependence, anxiety disorders, and prior ISH.

## Discussion

The current study is the largest study to date to examine the association between PTSD and non-fatal ISH among VHA patients, and the only such study to conduct gender-stratified analyses. We found that PTSD diagnosis was strongly associated with non-fatal ISH, even after matching on gender, age, and adjustment for marital status, baseline depression, substance abuse/dependence, anxiety disorder diagnoses and prior ISH among both male and female VHA patients. With regard to gender differences, the adjusted rate ratio observed among female Veterans was much larger in magnitude than the association observed for male Veterans (with non-overlapping confidence intervals); although the width of the confidence interval around the hazard ratio for women indicates that this association was imprecisely measured. Our finding with respect to non-fatal intentional self-harm among VHA patients is consistent with the only gender-stratified examination of PTSD and death from suicide among VHA patients, which found age-adjusted hazard ratios for the associations between PTSD and suicide of 1.8 in men and 3.5 in women (unadjusted for psychiatric comorbidities) (Ilgen et al. [2010]). The hazard ratios found in the current study are notably larger in magnitude, even after adjustment for relevant psychiatric confounders (3.2 in men and 16 in women), indicating that non-fatal suicidal behavior among women may be more strongly related to PTSD than prior work focusing on suicide has suggested.

We also found evidence of interaction between PTSD and depression diagnoses at any point in the study period in predicting non-fatal ISH, such that the rate of non-fatal ISH among people with PTSD and depression could not be explained by the individual rates among people with PTSD only, depression only, and the rate of ISH due to other causes. Contrary to work by Zivin et al. (Zivin et al. [2007]), who found that PTSD was protective against death from suicide in a sample of VHA patients with depression, our findings indicate that there is a synergistic effect when PTSD and depression co-occur, resulting in an increased number of non-fatal ISH events. In addition, we found that the IC was much larger for female VHA patients as compared to male VHA patients, highlighting the importance of gender-stratified examinations of VHA patients.

It is unclear why findings from the current study and those by Zivin et al. ([2007]) are disparate, given that there is no a priori reason to expect the direction of associations for non-fatal ISH and death from suicide to differ. It is interesting to note that Zivin et al. ([2007]) report a suicide rate of 68.2/100,000 person-years among VHA patients with PTSD and depression from 1999–2004,(Zivin et al. [2007]) while Ilgen et al. ([2010]) report a suicide rate of 68.6/100,000 person-years for all VHA patients with PTSD (not just those that have been diagnosed with depression) from 1999–2006 (Ilgen et al. [2010]). For both sets of findings to be accurate, patients in the Ilgen et al. ([2010]) study without a depression diagnosis would need to have had a suicide rate that was essentially the same as the suicide rate among people with a depression diagnosis (approximately 68/100,000). These inconsistencies highlight the need for more research that disentangles these important associations.

A few aspects of the current study should be kept in mind while interpreting our results. First, we restricted our outcome to non-fatal ISH events that resulted in hospitalization as outpatient treatment visits resulting from ISH events are not reliably coded in the data sources. Further, we were concerned that including outpatient suicide attempts may result in a systematic bias in the detection of ISH events (e.g., those receiving PTSD treatment at the VA may be more likely to report a less severe suicide attempt that required minimal or no treatment to their clinician). It is unclear how our results may generalize to non-fatal ISH events resulting in outpatient treatment only. Second, some people who attempt suicide may not seek medical attention after the event and some Veterans may not seek medical attention at a VHA hospital for acts of ISH. Our results may have been different if we were able to include all ISH events that occurred regardless of where or whether the patient was treated. Third, our data only includes VHA patients who were diagnosed and treated in Massachusetts, so it is possible that our results are not generalizable to Veterans who receive VHA treatment in other states. Likewise, the current study only includes VHA patients, so our results may not be generalizable to the larger population of Veterans. Finally, we were only able to adjust for certain demographic characteristics that were available within the VHA data (e.g., marital status); therefore our results may be impacted by unadjusted confounding by variables such as traumatic experiences, including those occurring during deployment, deployment and military demographics, service connection and socioeconomic status.

## Conclusions

Findings from the current study provide evidence of a strong association between PTSD and non-fatal ISH and indicate that when PTSD and depression co-occur, risk of non-fatal ISH is increased, especially for female VHA patients. Further, our results highlight the importance of gender-stratified examinations of VHA cohorts. The VHA system is the largest health care system in the United States with over 8.9 million Veterans enrolled in VA care in fiscal year 2013 (approximately 1/3 of all Veterans). (Department of Veterans Affairs OotA [2014]) Current initiatives for the prevention of suicidal behavior (including, but not limited to, suicide prevention coordinators at each VA hospital and a suicide crisis hotline) exist to aid Veterans in crisis. Our results suggest that initiatives aimed at treating PTSD and depression are an important focus for the prevention of ISH as well.

## Endnotes

^a^Pattern of results among the subsample without prior ISH was consistent with presented results. Results available from first author.

^b^Additional analyses examining the interaction between baseline depression diagnoses only and PTSD in predicting ISH in the full sample revealed a synergistic association as well. Results available from first author.

## Abbreviations

aIC: Adjusted interaction contrast
CI: Confidence interval
ICD-9-CM: International Classification of Diseases, 9th Edition, Clinical Modification
ISH: Intentional self-harm
PTSD: Posttraumatic stress disorder
PY: Person-years
VHA: Veterans Health Administration
